# Synthesis of Oxygen Heterocycles via Aromatic C-O Bond Formation Using Arynes

**DOI:** 10.3390/molecules200712558

**Published:** 2015-07-09

**Authors:** Hideto Miyabe

**Affiliations:** School of Pharmacy, Hyogo University of Health Sciences, 1-3-6, Minatojima, Chuo-ku, Kobe 650-8530, Japan; E-Mail: miyabe@huhs.ac.jp; Tel.: +81-78-304-3094; Fax: +81-78-304-2794

**Keywords:** oxygen heterocycles, arynes, synthesis, multi-component reaction

## Abstract

Most of the synthetic approaches to the benzo-fused heterocycles containing an oxygen atom have involved the use of phenol derivatives as a starting material. This review highlights the new synthetic approaches involving the aromatic C-O bond-forming process using arynes. The insertion of arynes into the C=O bond gives the unstable intermediates, [2 + 2] cycloaddition-type adducts, which can be easily converted into a variety of oxygen atom-containing heterocycles in a single operation. In this review, the syntheses of oxygen heterocycles, such as coumarin, chromene, xanthene, dihydrobenzofuran and benzofuran derivatives, via the insertion of arynes into the C=O bond of aldehydes or formamides are summarized.

## 1. Introduction

**Figure 1:**
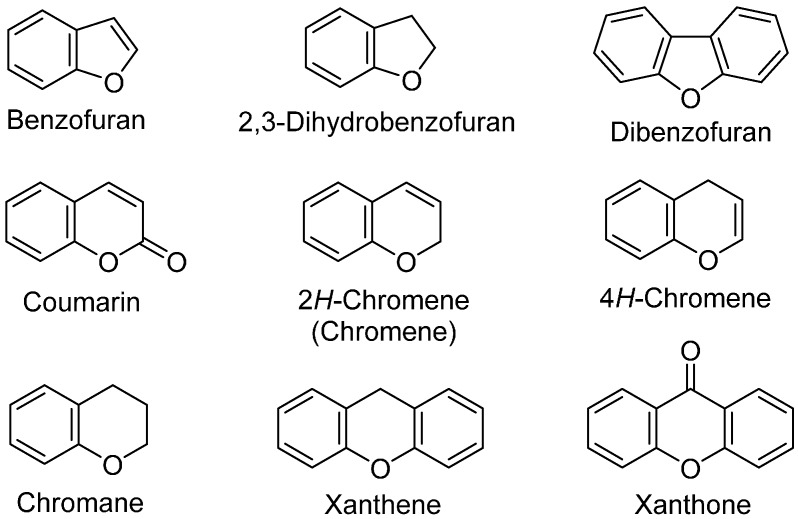
Benzo-fused heterocycles containing an oxygen atom.

Oxygen atom-containing heterocycles are an important class of the organic heterocyclic compounds. In particular, the benzo-fused oxygen heterocycles, in which oxygen heterocyclic ring is fused to benzene ring, are found as a key structural unit in natural products, pharmaceuticals and biologically active compounds (Figure 1) [1,2,3]. Therefore, benzo-fused oxygen heterocycles are of great synthetic interest. However, most of the synthetic approaches are based on the construction of oxygen heterocyclic ring from various phenol derivatives. Thus, the development of new approaches based on aromatic C-O bond formation continues to attract much interest.

**Figure 2:**
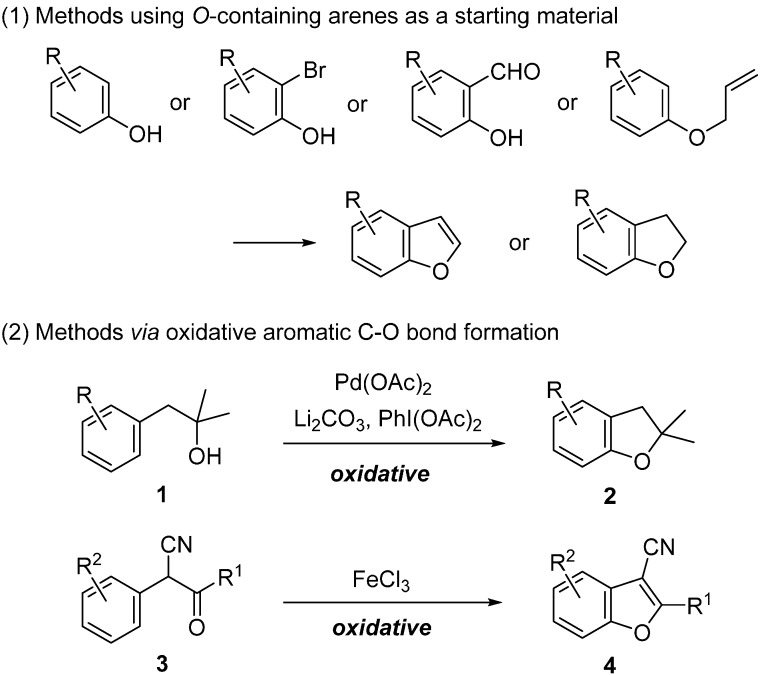
Synthetic approaches to benzofurans and 2,3-dihydrobenzofurans.

As an representative example, the synthetic approaches to benzofurans and 2,3-dihydrobenzofurans are shown in Figure 2. Many reported approaches have involved the use of oxygen atom-containing arenes such as phenol, 2-bromophenol or salicylaldehyde derivatives as a starting material [4,5,6,7,8]. As an approach based on aromatic C-O bond formation, the intramolecular transition metal-catalyzed ipso substitution of aryl halide with an alcohol moiety was studied [9,10,11,12,13,14,15,16]. More recently, the oxidative aromatic C-O bond forming methods were developed [17]. Yu reported the oxidative approach to dihydrobenzofurans **2** from alcohols **1** via Pd(II)-catalyzed and hydroxyl-directed C-H bond activation followed by C-O bond formation [18]. Zhao reported that FeCl_3_-mediated oxidative aromatic C-O bond forming cyclization of ketones **3** gave the benzofurans **4** [19].

**Figure 3:**
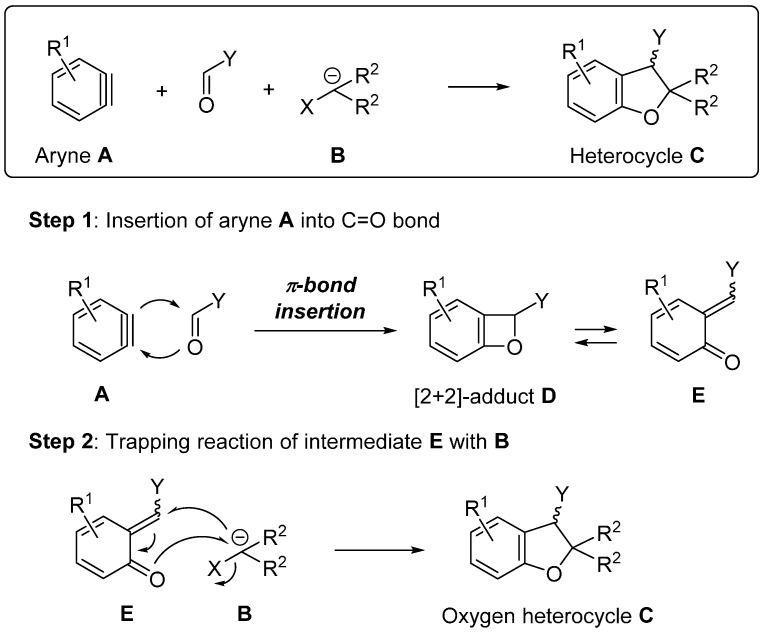
Method for aromatic C-O bond formation using arynes.

The use of arynes as the highly reactive intermediates in organic synthesis has attracted substantial attention [20,21,22,23,24,25,26,27]. The recent dramatic progress in aryne-based chemistry is summarized in the review articles [28,29,30,31,32,33,34,35,36,37,38,39,40,41,42,43,44,45]. The studies on the insertion of arynes into the π-bond are limited [37,46]. This review highlights the new synthetic approaches to oxygen heterocycles via the aromatic C-O bond-forming process based on the insertion of arynes into the C=O bond (Figure 3). When carbonyl compounds are employed, the insertion of aryne **A** into the C=O bond proceeds to give the unstable intermediate [2 + 2] cycloaddition-type adduct **D**, which isomerizes to the intermediate quinone methide **E** [47]. The subsequent trapping reaction of intermediate **E** with the reactant **B** having both nucleophilic and electrophilic sites gives oxygen atom-containing heterocycle **C** in a single operation.

**Figure 4:**
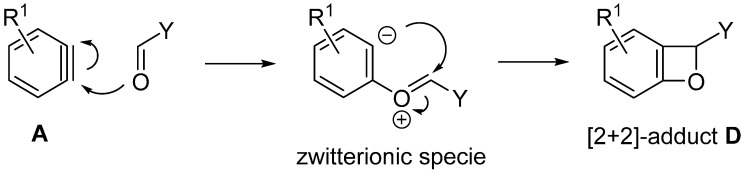
Stepwise mechanism.

As shown in Figure 4, the insertion of aryne **A** into the C=O bond giving the [2 + 2] cycloaddition-type adduct **D** is assumed to proceed via the stepwise [2 + 2] mechanism involving the zwitterionic specie as an intermediate.

## 2. Syntheses of Oxygen Heterocycles Using Insertion of Arynes into C=O Bond

### 2.1. Domino Reaction Starting from Insertion of Arynes into Aldehydes

**Scheme 1:**
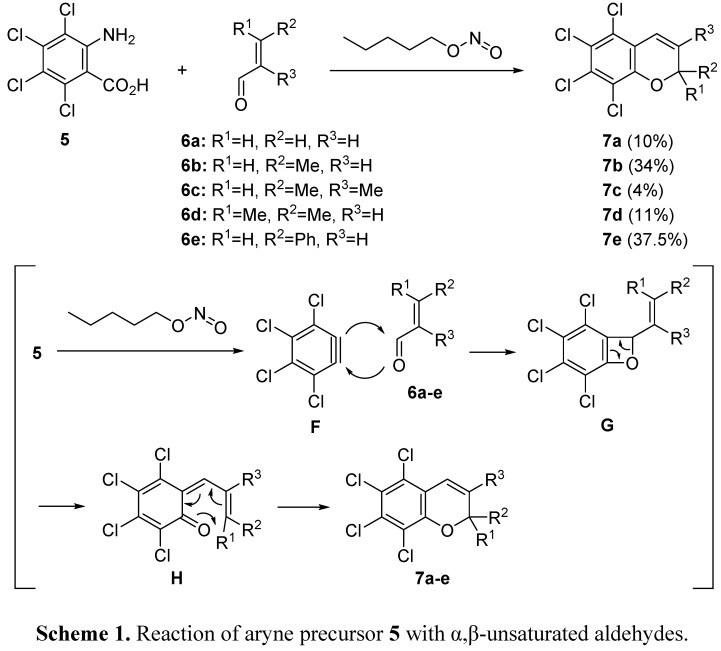
Reaction of aryne precursor **5** with α,β-unsaturated aldehydes.

Heaney studied the reaction of arynes with carbonyl compounds [48,49,50]. He reported a novel approach to the synthesis of 2*H*-chromenes based on the reaction of arynes with α,β-unsaturated aldehydes (Scheme 1) [48]. Tetrachloroanthranilic acid **5** was employed as an aryne precursor. In the presence of pentyl nitrite, treatment of **5** with α,β-unsaturated aldehydes **6a**–**e** gave 2*H*-chromenes **7a**–**e**. Although the yields obtained in the reaction with acrolein **6a**, 2,3-dimethylacrolein **6c** or 3,3-dimethylacrolein **6d** were not good, the use of crotonaldehyde **6b** and cinnamaldehyde **6e** led to the formation of 2*H*-chromenes **7b** and **7e** in the reasonable yields. Initially, aryne **F** is generated via the diazotization reaction of precursor **5** with pentyl nitrite. The insertion of aryne **F** into the C=O bond of aldehydes **6a**–**e** gives the formal [2 + 2]-type adduct **G**. The ring opening of [2 + 2]-type adduct **G** gives the intermediate quinone methide **H** which could undergo the intramolecular Diels-Alder reaction to afford 2*H*-chromenes **7a**–**e**.

**Scheme 2:**
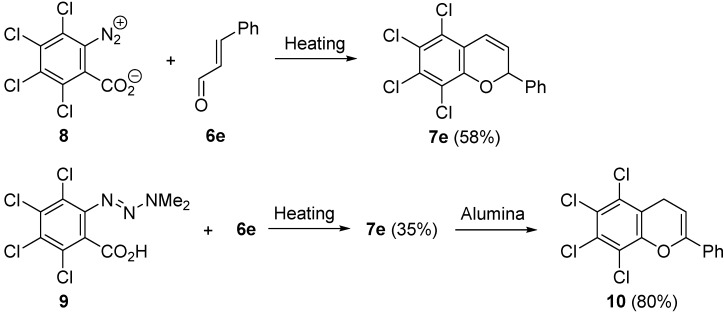
Reaction of precursors **8** and **9** with cinnamaldehyde **6e**.

Next, tetrachlorobenzenediazonium-2-carboxylate hydrochloride **8** and 3,4,5,6-tetrachloro-2-(3,3-dimethyltriazeno)benzoic acid **9** were employed as an aryne precursor (Scheme 2). When aryne precursor **8** was heated at 60 °C in chloroform containing an excess of cinnamaldehyde **6e**, 2*H*-chromene **7e** was obtained in 58% yield. Similarly, heating precursor **9** at 120 °C in tetrachloroethylene containing cinnamaldehyde **6e** gave 2*H*-chromene **7e** in 35% yield. Interestingly, the isomerization of 2*H*-chromene **7e** into 4*H*-chromene **10** was also observed. 4*H*-Chromene **10** was formed in 22% yield when the reaction of **9** with **6e** was carried out at 200 °C in the absence of a solvent. The effective isomerization of **7e** into **10** was achieved by the preparative layer chromatography using neutral alumina.

**Scheme 3:**
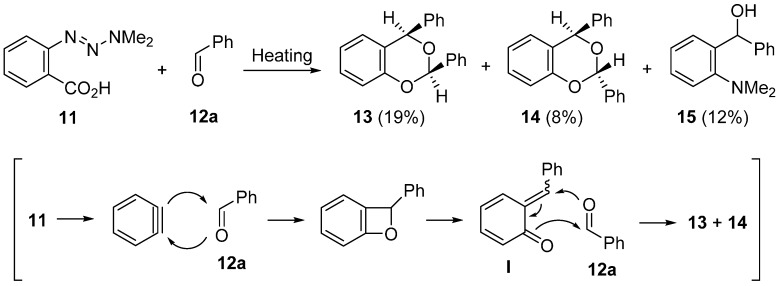
Reaction of aryne precursor **11** with benzaldehyde **12a**.

The reaction of benzyne with an excess amount of benzaldehyde was studied by Heaney and Nakayama, independently [50,51]. Nakayama reported that heating benzyne precursor **11** at 160 °C in benzaldehyde **12a** gave *cis*- and *trans*-2,4-diphenyl-1,3-benzodioxines **13** and **14** accompanied by the basic compound **15** (Scheme 3). Two isomeric cyclic products **13** and **14** are formed through the [2 + 2]-type reaction of benzyne, generated from precursor **11**, with the C=O bond of benzaldehyde **12a** followed by the trapping reaction of the intermediate quinone methide **I** with benzaldehyde **12a**. In contrast, 2-dimethylaminobenzhydol **15** is obtained as a result of the reaction of benzyne with HNMe_2_ generated from precursor **11**. Additionally. It is reported that the reaction of benzenediazonium-2-carboxylate with benzaldehyde **12a** in CH_2_Cl_2_ at 40 °C afforded *cis*-isomer **13** exclusively. Thus, the trapping reaction of quinone methide **I** with benzaldehyde **12a** would take place concertedly in *syn* fashion with the *endo* orientation.

**Scheme 4:**
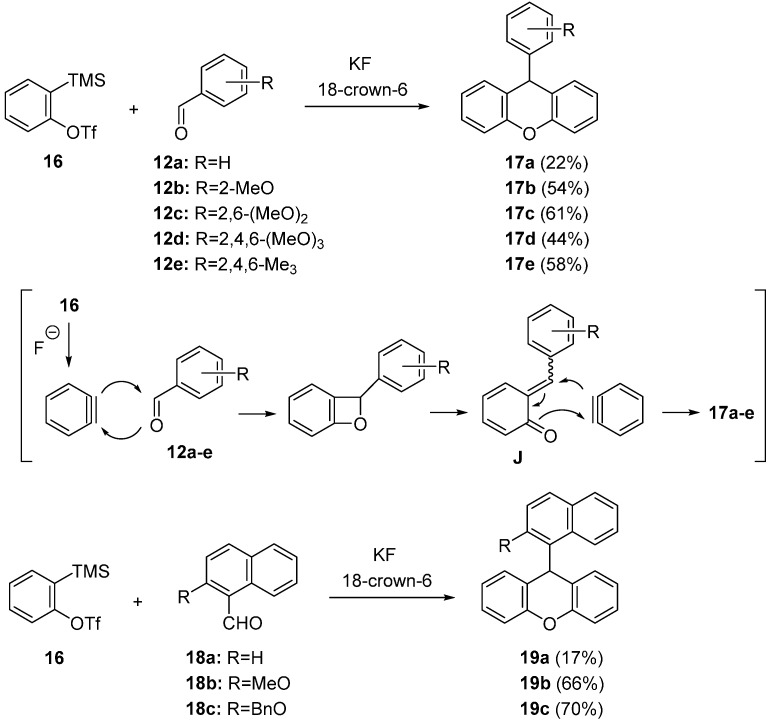
2:1-Coupling reaction of aryne precursor **16** with various aldehydes.

A straightforward method for the synthesis of xanthene derivatives was developed by Yoshida and Kunai’s group (Scheme 4) [52]. They reported that the 2:1 coupling reaction of two molar amounts of aryne and one molar amount of aryl aldehyde gave 9-arylxanthenes derivatives. The reaction was carried out in THF at 0 °C using *o*-trimethylsilylphenyl triflate **16** (0.45 mol) and aryl aldehydes **12a**–**e** (0.15 mol) in the presence of KF and 18-crown-6. The reaction of benzyne, generated from **16** and KF/18-crown-6, with variously substituted aryl aldehydes **12b**–**e** gave the 9-arylxanthenes **17b**–**e** in reasonable yields, although low yield was observed in the reaction with simple benzaldehyde **12a**. The reactions using naphthaldehydes **18a**–**c** or other substituted precursors are also reported. As shown in Scheme 4, the substituted naphthaldehydes **18b** and **18c** worked well to give the bulky xanthenes **19b** and **19c** in 66% and 70% yields, respectively.

### 2.2. Domino Reaction Starting from Insertion of Arynes into Formamides

**Scheme 5:**
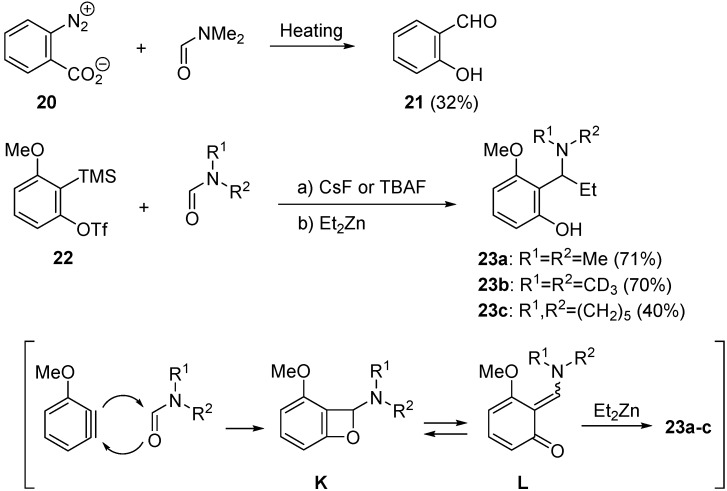
Reaction of arynes with formamides.

Domino reactions starting from the insertion of arynes into the C=O bond of formamides provide the new synthetic approaches to the benzo-fused heterocycles containing an oxygen atom. In 1965, Yaroslavsky reported that benzyne, generated from precursor **20**, reacted with *N*,*N*-dimethylformamide (DMF) to give salicylaldehyde **21** in 32% yield (Scheme 5) [53]. Recently, Miyabe studied the trapping reaction of the intermediates generated by the reaction of precursor **22** with formamides [54,55]. He reported that diethyllzinc trapped the intermediates **L** with good chemical efficiencies to give the aminophenols **23a**–**23c**. The mechanism involving the formation of formal [2 + 2]-type adducts **K** and quinone methides **L** is proposed.

**Scheme 6:**
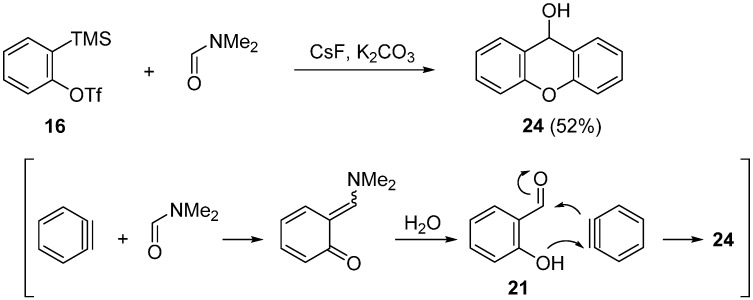
2:1-Coupling reaction of precursor **16** with DMF.

Okuma reported that the 2:1 coupling reaction of two molar amounts of benzyne and one molar amount of DMF gave 9-hydroxyxanthene (Scheme 6) [56]. In the presence of CsF and K_2_CO_3_, the reaction of precursor **16** (1.2 mol) with DMF (0.5 mol) in CH_3_CN at room temperature afforded 9-hydroxyxanthene **24** in 52% yield. 9-Hydroxyxanthene **24** would be formed by the reaction of salicylaldehyde **21** with benzyne. The formation of xanthene and xanthone through the disproportionation of 9-hydroxyxanthene **24** is also reported.

**Scheme 7:**
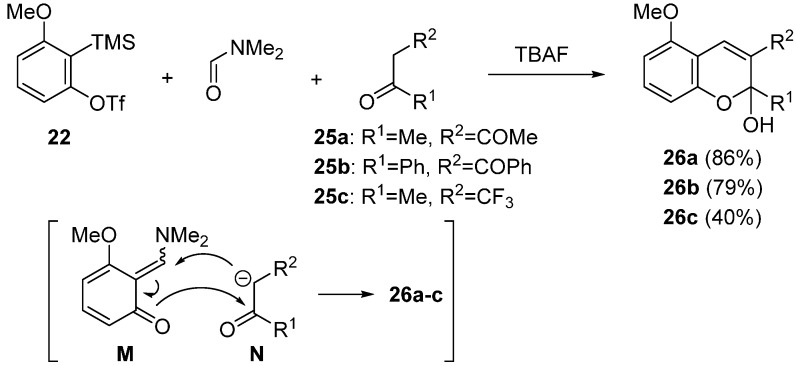
Synthesis of 2*H*-chromene derivatives.

A method for preparing 2*H*-chromene derivatives was developed by Miyabe (Scheme 7) [57]. Three-component coupling reaction leading 2*H*-chromenes **26a**–**c** was achieved by the use of active methylene compounds **25a**–**c** as a nucleophile for trapping the unstable intermediate **M**. In the presence of anhydrous TBAF as fluoride ion source, treatment of precursor **22** with acetylacetone **25a** in DMF at room temperature gave the 2*H*-chromene **26a** in 86% yield. Similarly, the bulky 1,3-diketone **25b** bearing two phenyl groups and the acetone **25c** having an α–CF_3_ group acted as a nucleophile trapping quinone methide **M** to give the corresponding 2*H*-chromenes **26b** and **26c** in 79% and 40% yields, respectively.

**Scheme 8:**
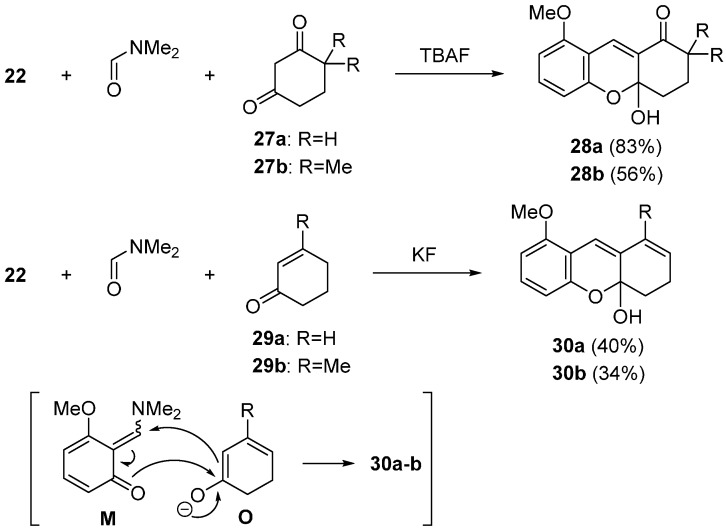
Synthesis of tricyclic 2*H*-chromene derivatives.

The tricyclic 2*H*-chromene derivatives **28a** and **28b** were obtained when cyclic 1,3-diketones **27a** and **27b** were employed as a nucleophile (Scheme 8) [57]. Three-component coupling reaction with **27a** at room temperature produced tricyclic compound **28a** in 83% yield. In the case of unsymmetrical diketone **27b**, the compound **28b** was obtained as a major regioisomer. The formation of tricyclic 2*H*-chromene derivatives was also observed when cyclohexenone derivatives **29a** and **29b** were employed as a nucleophile [57]. In the presence of KF, the reaction using precursor **22** and cyclohexenone **29a** was carried out in DMF at 80 °C to give the tricyclic product **30a** in 40% yield. Under the similar reaction conditions, the desired compound **30b** was obtained in 34% yield even when bulky nucleophile **29b** was employed. This transformation would involve the trapping reaction of the intermediate **M** with anions **O** generated from cyclohexenones **29a** and **29b**.

**Scheme 9:**
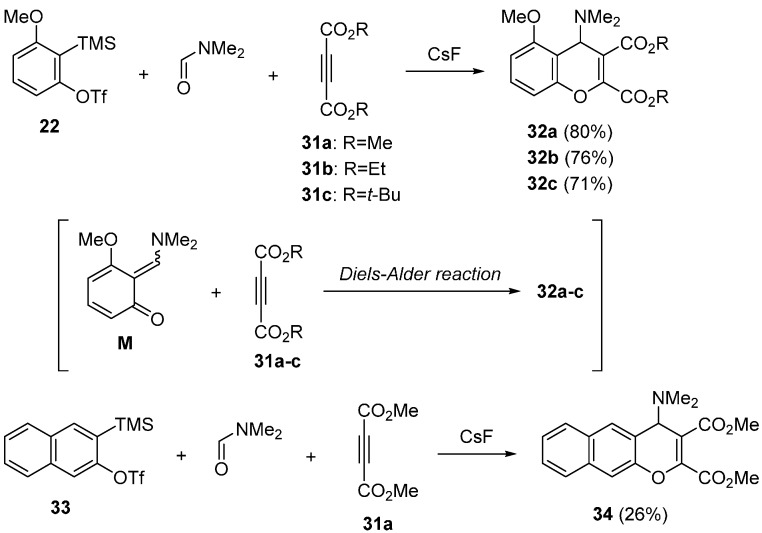
Synthesis of 4*H*-chromene derivatives.

Miyabe reported the synthesis of 4*H*-chromene derivatives (Scheme 9) [58]. Three-component coupling reaction involving the hetero Diels-Alder reaction of the transient intermediate **M** with dienophiles was investigated. In the presence of CsF, the reaction using precursor **22** and acetylenedicarboxylic acid dimethyl ester **31a** in DMF proceeded effectively at 25 °C. The 4*H*-chromene **32a** was obtained in 80% yield after being stirred 2 h. The heating activation at 50 °C accelerated the reaction to give **32a** in 79% for 15 min. Under analogous reaction conditions, diethyl ester of acetylenedicarboxylic acid **31b** has shown the good reactivity. Moreover, bulky acetylenedicarboxylic acid di-*tert*-butyl ester **31c** worked well to give the product **32c** in 71% yield. The reaction of aryne precursor **33** was also reported.

**Scheme 10:**
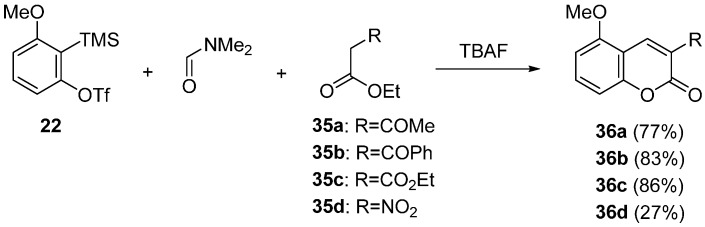
Synthesis of coumarin derivatives.

The synthesis of coumarin derivatives was studied by Miyabe and Yoshida, independently [57,59]. Miyabe reported three-component coupling reaction using β–keto esters as a nucleophile trapping the intermediate quinone methide (Scheme 10) [57]. When β–keto ester **35a** was employed, coumarin **36a** was synthesized in 77% yield. High chemical yields were observed in the reactions using β–keto ester **35b** having a phenyl group or diethyl malonate **35c**. In contrast, the reaction of ester **35d** having a nitro group proceeded, albeit with relatively lower yield.

**Scheme 11:**
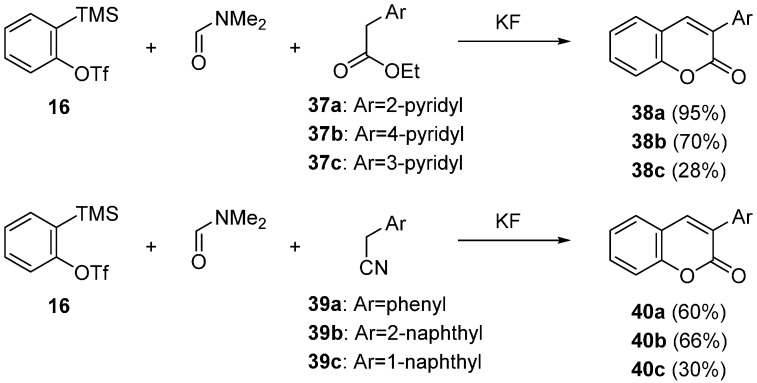
Synthesis of coumarin derivatives having an aryl group.

Yoshida studied three-component coupling reaction for the synthesis of coumarin derivatives [59]. The efficient method for preparing the coumarins substituted an aryl group at 3 position was reported (Scheme 11). In the presence of KF, the reaction using acetates **37a**–**c** having an aryl group was carried out in DMF at 80 °C to give the coumarins **38a**–**c**. Interestingly, acetonitriles **39a**–**c** having aryl group acted as a nucleophile under similar conditions. The reaction using precursor **16** and phenylacetonitrile **39a** in DMF proceeded at 80 °C to afford the coumarin **40a** in 60% yield after being stirred 6.5 h. Although the reaction of bulky 1-naphthylacetonitrile **39c** resulted in a low yield, 2-naphthylacetonitrile **39b** effectively participated in the reaction to give **40b** in 66% yield.

**Scheme 12:**
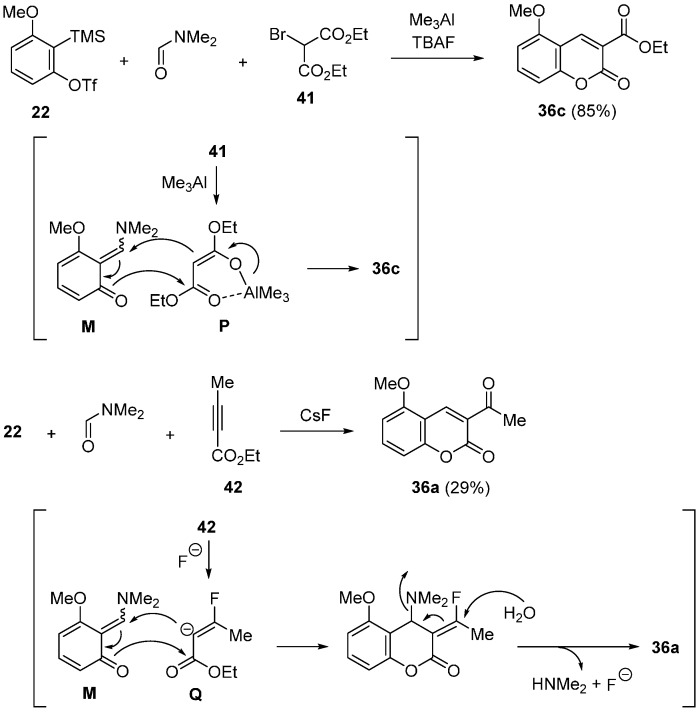
Synthesis of coumarin derivatives using reactants **41** and **42**.

Coumarin **36c** was effectively synthesized by the use of debrominated metal enolate **P**, which was *in situ* generated by a combination of α-bromomalonate **41** and Me_3_Al (Scheme 12) [60]. In the presence of anhydrous TBAF, precursor **22** was reacted with **41** and Me_3_Al in DMF at room temperature to give the desired coumarin **36c** in 85% yield. Interstingly, the formation of coumarin **36a** was observed when ethyl 2-butynoate **42** was used [58]. In this transformation, the anion **Q** would be generated by the addition of fluoride ion to butynoate **42**. The trapping reaction of quinone methide **M** with anion **Q** would lead to the formation of coumarin **36a**.

**Scheme 13:**
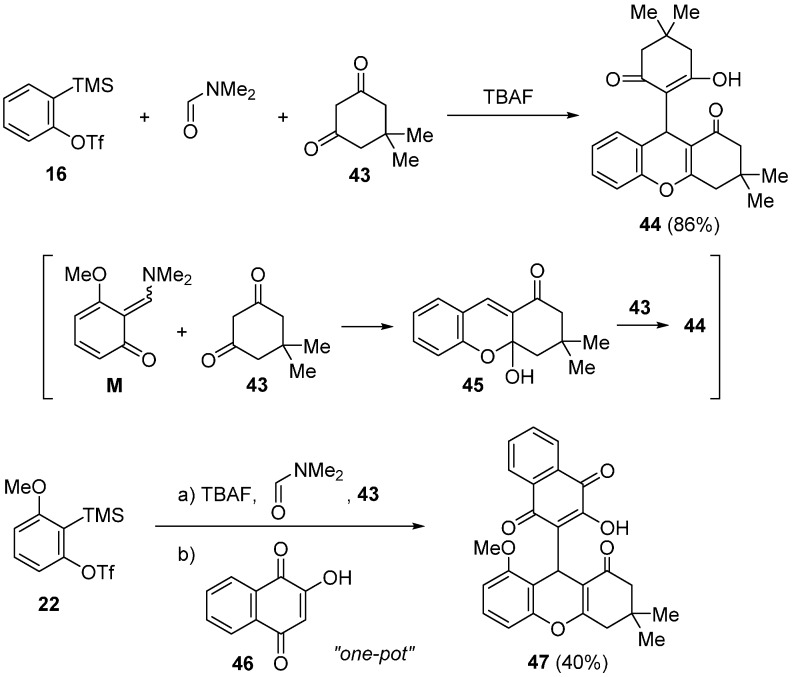
Multicomponent coupling reaction.

Three-component coupling reaction for preparing 2*H*-chromenes as shown in Scheme 7 was successfully applied to four-component coupling reaction for the convenient synthesis of xanthene derivatives (Scheme 13) [57]. In the presence of anhydrous TBAF, treatment of aryne precursor **16** (1.0 equiv.) with dimedone **43** (2.5 equiv.) in DMF at room temperature gave xanthene derivative **44** in 86% yield. In this transformation, three-component coupling product 2*H*-chromene **45** reacted again with an excess amount of dimedone **43** to give **44** in one-pot. Four-component coupling reaction using two different 1,3-diketones also proceeded by a one-pot procedure. When 2-hydroxy-1,4-naphthoquinone **46** was used as a nucleophilic reactant, the direct one-pot synthesis of xanthene derivative **47** from precursor **22** was achieved [61]. These transformations involve the three C-C and two C-O bond-forming processes under mild neutral conditions.

**Figure 5:**
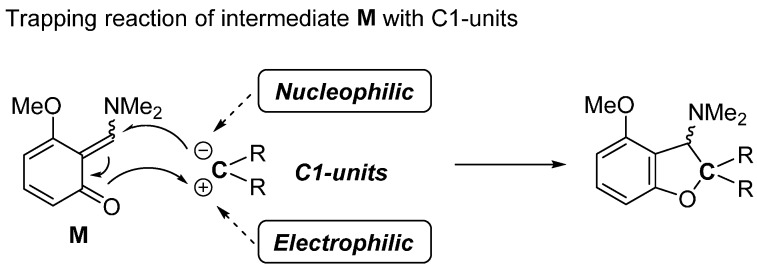
Method for the synthesis of dihydrobenzofurans and benzofurans.

For the construction of the five-membered oxygen heterocyclic rings such as dihydrobenzofurans and benzofurans, the intermediate quinone methide **M** must be trapped with C1-units having a nucleophilic and electrophilic carbon atom (Figure 5).

**Scheme 14:**
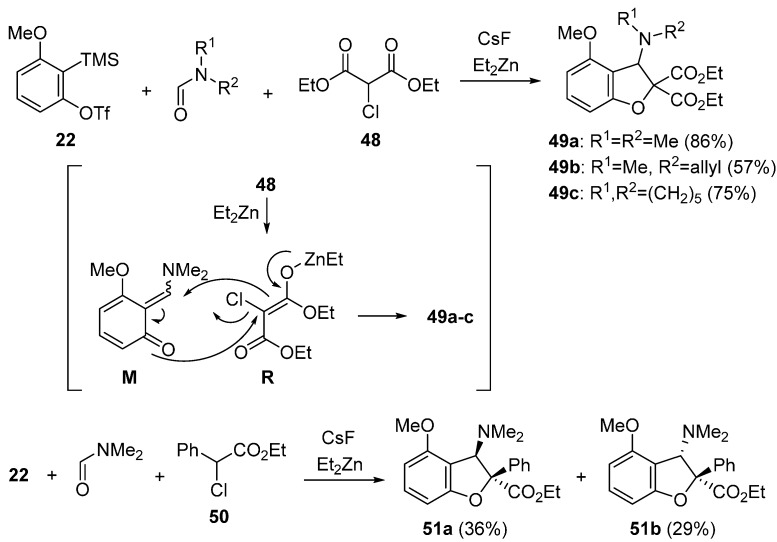
Synthesis of 2,3-dihydrobenzofurans having a dimethylamino group.

Miyabe used α-halogenated enolates as a nucleophilic and electrophilic C1-unit for trapping the intermediate **M** (Scheme 14) [60,62]. He reported that the desired α-halogenated enolate **R** was effectively prepared by a combination of α-chloromalonate **48** and Et_2_Zn. In the presence of CsF and Et_2_Zn, treatment of precursor **22** with α-chloromalonate **48** in DMF at −40 °C to room temperature gave 2,3-dihydrobenzofuran **49a** in 86% yield. Under similar reaction conditions, dihydrobenzofuran **49b** having *N*-methyl and *N*-allyl groups was obtained from unsymmetrical formamide. Additionally, 1-formylpiperidine worked well to give 2,3-dihydrobenzofuran **49c**. Moreover, three-component coupling reaction using ethyl α-chlorophenylacetate **50** took place to afford two diastereomers **51a** and **51b** in acceptable yields.

**Scheme 15:**
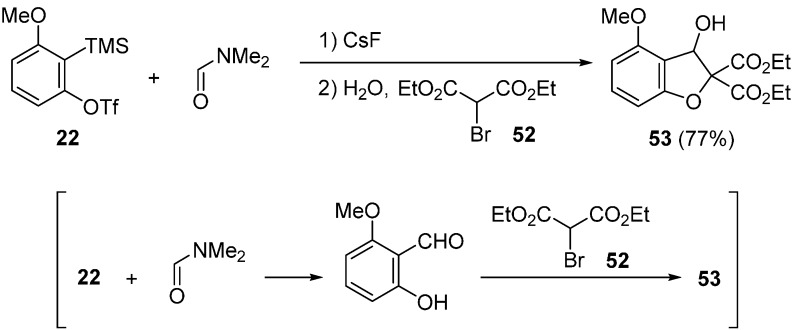
Synthesis of 2,3-dihydrobenzofuran having a hydroxy group.

The synthesis of 2,3-dihydrobenzofuran **53** having a hydroxy group was also reported (Scheme 15) [60]. When α-bromomalonate **52** was used as a C1-unit together with a small amount of water, the desired dihydrobenzofuran **53** was obtained in 77% yield instead of dihydrobenzofuran **49a** having a dimethylamino group.

**Scheme 16:**
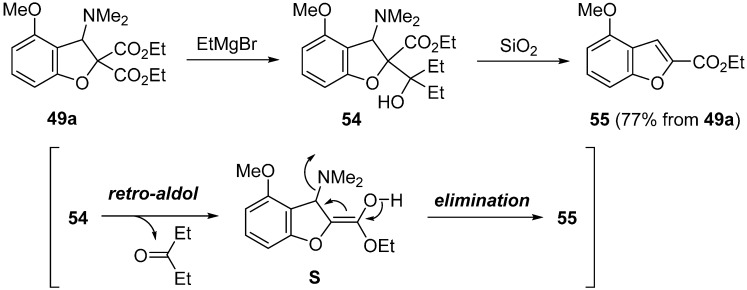
Conversion of 2,3-dihydrobenzofuran **49a** into benzofuran **55**.

The conversion of 2,3-dihydrobenzofurans into benzofurans was studied (Scheme 16) [60]. Treatment of dihydrobenzofuran **29a** with 2.5 equivalents of EtMgBr in THF at −40 °C to room temperature followed by SiO_2_ in AcOEt at room temperature gave benzofuran **55** in 77% yield. This transformation was carried out by one-pot procedure without the isolation of adduct **54**. The disered benzofuran **55** is formed via the retro-aldol type reaction of **54** followed by the elimination of a dimethylamino group of intermediate **S**.

**Scheme 17:**
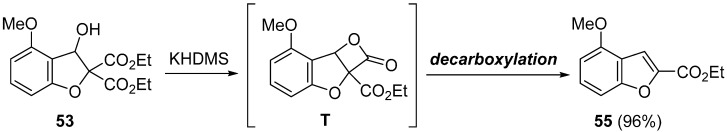
Conversion of dihydrobenzofuran **53** having a hydroxy group.

As an alternative approach for the synthesis of benzofurans, another effective transformation of 2,3-dihydrobenzofuran **53** having a hydroxy group into benzofuran **55** was reported (Scheme 17) [60]. This transformation would involve the decarboxylation of the cyclic intermediate **T** [63]. The base had an impact on the chemical efficiency of this transformation. LiHMDS and NaHMDS were less effective. When KHMDS was employed as a base in THF at −40 °C, benzofuran **55** was obtained in 96% yield.

**Scheme 18:**
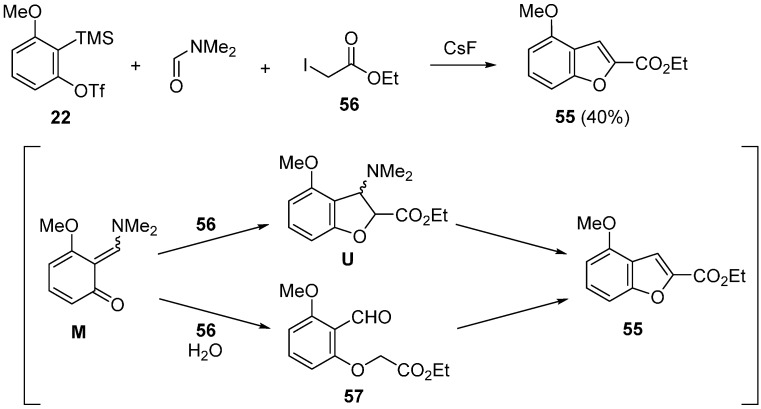
Direct synthesis of benzofuran.

Direct synthesis of benzofurans from aryne precursors was also investigated [60,62]. The method using ethyl iodoacetate **56** as a C1-unit is shown in Scheme 18 [60]. In the presence of CsF, the reaction of precursor **22** with **56** was carried out in DMF at 100 °C to give the benzofuran **55** in 40% yield. When the same reaction was carried out at room temperature, simple *O*-alkylated product **57** was formed. Additionally, the formation of benzofuran **55** was observed in heating *O*-alkylated product **57** at 100 °C. Based on these results, two possible reaction pathways are proposed. As a direct pathway, benzofuran **55** is obtained from the intermediate **U**, which is generated by the trapping reaction of quinone methide **M** with ethyl iodoacetate **56**. Another pathway is the formation of benzofuran **55** from *O*-alkylated product **57**.

**Scheme 19:**
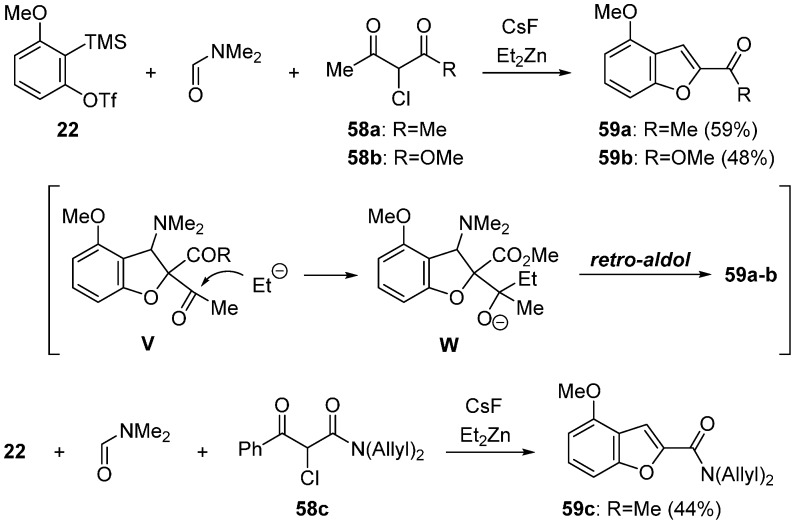
Direct synthesis of 2,3-benzofurans using Et_2_Zn.

The direct one-pot synthesis of benzofurans through the retro-aldol type reaction was reported (Scheme 19) [62]. For this transformation, the α-halogenated active methines having a ketone group were used as a C1-unit, since the reaction of ketone moiety with Et_2_Zn leads to the retro-aldol type process. In the presence of CsF, treatment of active methines **58a** and **58b** with Et_2_Zn and precursor **22** at −60 °C to room temperature led to the direct formation of benzofurans **59a** and **59b**. This transformation proceeds via the addition of an ethyl anion to a ketone group of **V** followed by the retro-aldol type reaction of **W**. The methine **58c** having a bulky phenyl ketone group worked well to give benzofuran **59c**.

## 3. Concluding Remarks

The recent aryne-based chemistry has achieved some remarkable success. Particularly, the insertion of arynes into the C=O bond has been studied as a powerful method for preparing the benzo-fused oxygen heterocycles. These aromatic C-O bond forming reactions proceed under mild transition metal-free conditions. Moreover, synthetic strategies involving multicomponent coupling reaction offer the advantage of multiple carbon-carbon and/or carbon-heteroatom bond formations in a single operation. In addition to the insertion of arynes into various element-element σ-bonds, the corresponding π-bond insertion disclosed a broader aspect of the utility of arynes in synthetic organic chemistry. This domain offers opportunities for further exploration with intriguing possibilities in aryne chemistry. I hope that this review will inspire new creative contributions by organic chemists.
